# Psychiatry

**Published:** 1905-08-12

**Authors:** 


### PSYCHIATRY.

**Tuberculosis in Asylums for the Insane.**—The subject of tuberculosis in asylums still remains a serious question. It has been discussed alike from clinical, pathological, etiological, and preventive standpoints by a number of recent writers, including Dr. C. Floyd Haviland, of New York, the Tuberculosis Committee of the Medico-Psychological Association, whose revised report by Dr. Chapman, a statistical expert and practical alienist, published in 1903,[1] constitutes the standard document based on official medical statistics of the asylums of the United Kingdom, and by many others. In the most recent issue of the *Journal of Mental Science*,[2] Dr. Menzies refers to the question of how to reduce the death-rate of tuberculosis among inmates of asylums. "In the eighties and early nineties," he says, "we did not look with alarm on a high tubercular death-rate. Until about this time the asylum rate seems to have been gradually falling, although not in corresponding ratio to that of the sane population, but since then it has tended to rise." That some association exists between tuberculosis and insanity is now well known, for the tuberculous diathesis in the parentage predisposes to congenital idiocy, imbecility, and the more gross degenerative insanities of the young. And the mean or average populations of asylums, counted as admissions, have from long experience and observation been known to be more prone to contract phthisis and die of phthisis than the average sane population. "In 1903," says Dr. Menzies, "3·2 per cent. of the admissions to Cheddleton Asylum showed evidence of early or marked tuberculosis. Some of them were epileptic, some imbecile, some general paralytic, and many (at least half) of the total number were cases of recent acute maniacal and depressive insanity lasting from a few days to a few weeks. These latter cases afforded an appreciable percentage of primary adult insanity as compared with the larger and more prevalent tuberculous etiology of the idiot and imbecile classes before mentioned. The epileptics infect their ward," argues Dr. Menzies, "the general paralytics infect the physically infirm in the infirmary. But it is chiefly the chronic residue (of patients) which cause the trouble, for they infect many others around them, and especially those who are working in dusty places, the upholsterer's or stonemason's shop, or the bakehouse." Each chronic patient thus employed becomes a "focus of infection," and the tubercular death-rate of the asylum is "ten times as high as that of the general population at corresponding ages outside, and which is in a position of great inferiority," adds Dr. Menzies, "from a sanitary standpoint. . . . A nice state of affairs, and one wonders how it could have stood so long. There are a good many points in asylum administration in which our ideas have become hoary and crusted." The remedial measures proposed by Dr. Menzies are as follows:—(*a*) The temperature of every fresh admission should, for the first week, be taken in the morning, and at 2, 4, and 6 P.M. In this way the rise of temperature which occurs in some part of the 24 hours will be detected. The mouth is not a true index for temperature; "either the rectum, or, in sensible males, the issuing stream of urine should be used." All cases which present any rise are gone into more fully, and, if no other satisfactory explanation is forthcoming, like constipation, sepsis, etc., are removed to the phthisical ward and set aside for tuberculin injection when the temperature has become normal. Meanwhile they are to be treated by strict rest in bed. (*b*) Physical signs of incipient phthisis in the chest, however slight, should be regarded as positive evidence until tuberculin has disproved them. One cannot help noticing, he says, how much more common "short wind" is in the athletic members of tubercular families than in others of more healthy heredity. (*c*) The tubercular *facies*—an appearance of active anæmia combined with the special familiar features of one or other of the tubercular diatheses—should be looked for. (*d*) Tuberculin injections should be tried in moderate doses—1 to 5 milligrammes—and if the statement is true that these doses when injected in the absence of fever are harmless, it is evident that its employment in all admissions will settle the question with ease and certainty. (*e*) The weight of patients on admission should be determined frequently (not less than once a week); loss of weight should lead to the suspicion of phthisis if the condition was not due to the insanity itself. Dr. Menzies then proceeds to refer to the importance of segregation of phthisical patients in separate hospital blocks (male and female), away from the main building; and insists on the importance of good ventilation, with free access of the open air into the day-rooms, sitting-rooms, and dormitories and of providing suitable upholstery of the rooms, adequate heating arrangements and generous diet, while the use of spitting-mugs containing antiseptics should be enforced. Milk supplied to the patients should be previously boiled, or, rather, "Pasteurised," and tuberculous cows strictly eliminated from the cattle stock of the asylum farm.

**Tent Life for the Tuberculous Insane.**—The subject of tent life for the tuberculous insane has received considerable attention in the United States, and, having been tried practically during the last three years, its success has stimulated its wider adoption. In 1903 Dr. C. Floyd Haviland,[3] of New York, called attention to, and recorded the results obtained in, this new development of therapeutics. A scheme of "tent life" was inaugurated at the Manhattan State Asylum, Ward's Island, New York City, by Dr. A. E. Macdonald in 1902. The working of this system of isolation and open-air treatment is stated to have been attended with the most gratifying results during the first and subsequent years during which it has been in operation. The following is a brief *résumé* of the methods. Forty tuberculous insane patients, including in this number cases of miliary tuberculosis and pulmonary tuberculosis, began "tent life." Two large tents were employed, each being 14 feet high and having a capacity of 20 beds so as to allow of 40 square feet of floor space per bed. These tents were erected upon elevated dry ground surrounded by abundant trees and constantly swept by breezes from the East River, which they overlooked. The ground was cleared of all vegetation beneath each tent, and a board floor was constructed about 18 inches above the ground. The floor being made in sections, could be taken up and exposed to direct sunlight as occasion required. In pleasant weather one side of the tent was kept constantly open to the pure air outside, and at all times ample ventilation was afforded by large ventilators placed near the top of the tent at either end. Close by these tents were erected several small auxiliary tents, including two dining tents, a clothing tent, and tents for the use of attendants and nurses. Other conveniences were also erected at various distances for the use of the camping patients. All food was supplied from the main kitchen and eaten in the dining tents by those not confined to bed. The patients received frequent baths, and strict personal cleanliness was observed. The floors of the tent were regularly scrubbed with warm water containing carbolic acid, the broom being rarely used. All linen was thoroughly disinfected before being sent to the laundry. The expectoration cups and utensils constantly contained a 2-per-cent. solution of creolin, and in no circumstances was the sputum allowed to become dry, The atmosphere was sprayed hourly with a solution of the following:—Guiacol, 150 grammes; eucalyptol, 120 grammes; carbolic acid, 90 grammes; menthol, 60 grammes; thymol and oil of caraway seed, of each 30 grammes; and rectified spirit, 2,500 grammes. This spray, apart from its antiseptic properties, was found to be obnoxious to insects. Asepsis was thus observed in every detail, while under this plan of treatment complete isolation from the rest of the asylum population was secured. Four meals were served daily, the diet being ample, nutritious, and varied, and consisting of milk, eggs, meat, cocoa, beef-tea, fresh fruit, and vegetables. Particular attention was given to the free use of milk from cows on the asylum farm, which were examined and found to be free from tuberculosis. Only symptomatic medicinal treatment was resorted to, reliance for the improvement of patients being placed chiefly upon the hygienic measures employed. Owing to the success of this plan of treatment during the summer months it was continued on a smaller scale—one large tent with the necessary auxiliary tents, accommodating 20 of the more active patients—throughout the year. As winter approached heating arrangements were provided by erecting two large heat-radiating stoves, one at each end of the tent, while an ordinary grate-stove, with pipe and ventilating attachment, was also erected, and a coal-fire was kept burning constantly in it during the winter. All danger from over-heating or from fire was obviated by having the parts of the grate and of the pipe which came in contact with wood or canvas thickly covered with asbestos, while there was no difficulty in maintaining an equable temperature in the tent throughout the winter. During one year, says Dr. Haviland, 81 patients were given the benefits of tent life. The average death-rate among tuberculous inmates, which in recent years had been 14 per cent. in the Manhattan State Asylum, was found to fall to 8½ per cent. among the patients who led a "tent life." A chart of the progress in health of the patients demonstrated that during their camp life many of them greatly improved. Of the 81 cases 55 showed an increase in body weight, the average gain being 6½ lb. at the end of the year. The patients who died were all in an advanced stage of the disease when admitted. Appetite and assimilation of food were improved, pyrexia was diminished, and night sweats were notable by their absence. The encouraging results prompted Dr. Haviland to continue the system during a second and a third year (1904), with very gratifying results. Without being unduly enthusiastic, says Dr. Haviland, it may be stated that tent life pursued under the conditions described gave better prospects for health and improvement and more advantages than other practical plans of treatment for the tuberculous insane.

[1] Jour. of Mental Sci., 1903. [2] Ibid., July 1905. [3] Amer. Jour. of Med. Sci., 1903.

*(To be concluded.)*

